# The prognostic value of intraoperative HRV during anesthesia in patients presenting for non-cardiac surgery

**DOI:** 10.1186/s12871-023-02118-9

**Published:** 2023-05-09

**Authors:** Jiahe Niu, Yonghao Lu, Ruikun Xu, Fang Fang, Shikai Hong, Lexin Huang, Yajun Xue, Jintao Fei, Xuegong Zhang, Boda Zhou, Ping Zhang, Rui Jiang

**Affiliations:** 1 Department of Automation, Tsinghua University, No. 168 Li Tang Road, Changping District, Beijing, 100084 China; 2 Department of Cardiology, School of Clinical Medicine, Beijing Tsinghua Changgung Hospital, Tsinghua University, No. 168 Li Tang Road, Changping District, Beijing, 102218 China

**Keywords:** Anesthesia, HRV, Machine learning, Surgery, Clinical outcome

## Abstract

**Objective:**

To examine the prognostic value of HRV measurements during anesthesia for postoperative clinical outcomes prediction using machine learning models.

**Data sources:**

VitalDB, a comprehensive database of 6388 surgical patients admitted to Seoul National University Hospital.

**Eligibility criteria for study selection:**

Cases with ECG lead II recording duration of less than one hour were excluded. Cases with more than 20% of missing HRV measurements were also excluded. A total of 5641 cases were eligible for the analyses.

**Methods:**

Six machine learning models including Logistic Regression (LR), Support Vector Machine (SVM), Random Forest (RF), Gradient Boosting Decision Trees (GBT), Extreme Gradient Boosting (XGB), and an ensemble of the five baseline models were developed to predict postoperative clinical outcomes. The prediction models were trained using only clinical information, and using both clinical information and HRV features, respectively. Feature importance based on the SHAP method was used to assess the contribution of the HRV measurements to the outcome predictions. Subgroup analysis was also performed to evaluate the risk association between postoperative ICU stay and various HRV measurements such as heart rate, low-frequency power (LFP), and short-term fluctuation DFA ${\alpha }_{1}$.

**Result:**

The final cohort included 5641 unique cases, among whom 4678 (83.0%) cases had ages over 40, 2877 (51.0%) were male, 1073 (19.0%) stayed in ICU after surgery, 52 (0.9%) suffered in-hospital death, and 3167(56.1%) had a total length of hospital stay longer than 7 days. In the final test set, the highest AUROC performance with only clinical information was 0.79 for postoperative ICU stay, 0.58 for in-hospital mortality, and 0.76 for the total length of hospital stay prediction. Importantly, using both clinical information and HRV features, the AUROC performance was 0.83, 0.70, and 0.76 for the three clinical outcome predictions, respectively. Subgroup analysis found that patients with an average heart rate higher than 70, low-frequency power (LFP) < 33, and short-term fluctuation DFA ${\alpha }_{1}$ < 0.95 during anesthesia, had a significantly higher risk of entering the ICU after surgery.

**Conclusion:**

This study suggested that HRV measurements during anesthesia are feasible and effective for predicting postoperative clinical outcomes.

**Supplementary Information:**

The online version contains supplementary material available at 10.1186/s12871-023-02118-9.

## Key points

Question: Whether intraoperative HRV during anesthesia in patients presenting for non-cardiac surgery could have prognostic value.

Findings: This study suggests that HRV measurements during anesthesia are feasible and effective for predicting postoperative clinical outcomes.

Meaning: Intraoperative HRV during anesthesia in patients presenting for non-cardiac surgery may be used to predict prognosis in the future.

## Introduction

Anesthesia is a medical treatment used to prevent pain during surgical procedures. The adverse effects of the complex stress response could lead to serious complications and possibly, the risk of death. Early retrospective studies reported that the overall risk of death from general anesthesia is possibly at least 1 per 40000 anesthetics [1] while a perioperative mortality rate of 16 per 10000 anesthetics was reported in a later investigation [2]. Given the mortality rate of anesthetic patients undergoing surgery, it is reasonable to speculate possible predictors of perioperative clinical outcomes to improve survivals, reduce the length of ICU stay, and ultimately provide special care for poor-outcome patients. Recent studies suggested that there is a strong association between HRV and the nociception–analgesia balance during general anesthesia under surgery [3, 4].

Heart rate variability (HRV) is defined as the changes in time intervals between consecutive heartbeats (R-R intervals). The variability of heart rate is controlled by the autonomic nervous system (ANS) including the sympathetic nervous system (SNS) and the parasympathetic nervous system (PNS). HRV has been found to reflect the balance between the two nervous systems [5] and is frequently introduced as mirroring imbalances within the autonomic nervous system. The measurements of HRV can be easily obtained from long-term ($\ge 24 hours$) or short-term ($\le 5 minutes$) electrocardiogram (ECG) recordings, as a result, HRV has been considered as a low-cost and non-invasive method for quantitative measurements of autonomic activity [6]. Recent studies identified HRV as a promising marker for mortality risk assessment and patients’ outcome prediction [7, 8]. The reliable measurement of autonomic conditions offers the opportunity to detect autonomic dysfunction-related illnesses such as myocardial infarction, trauma, sepsis, and brain injury [9–12].

There have been investigations on the association of HRV parameters and the incidence of major adverse cardiac and cerebrovascular events such as cardiac arrest, traumatic brain injury, congestive heart failure, and myocardial infarction [12–15]. Abnormal HRV was found to be strongly associated with congestive heart failure in old adult population groups [16]. Both classical and recent experiments show that patients with decreased HRV significantly increased the risk for poor clinical outcomes, including death with or without congestive heart failure [13–15, 17, 18]. Meta-analysis investments are also implemented to further explain the relationship between HRV and diagnosis [19]. Therefore, HRV has shown a promising result as a predicting marker for cardiovascular-related disorders. However, for patients undergoing general or regional anesthesia, the HRV parameters under such states present fairly different characteristics, which makes it difficult to be interpreted. Some machine learning methods were used to predict the human state with the HRV, but those methods and the medical explanations were not comprehensively introduced [20, 21]. As a result, HRV during anesthesia lacks an understandable explanation, and the study on the prognostic values of intraoperative HRV during surgery remains an open research problem.

This study aims to investigate the aspects of HRV measurements obtained from admissions in patients undergoing non-cardiac surgery from the VitalDB database and to examine whether HRV during anesthesia could be used to predict clinical outcomes such as in-hospital mortality, postoperative ICU stays, and total length of hospital stays.

## Methods

### Data collection and preprocessing

We conducted a comprehensive study to investigate the potential relationship between intraoperative HRV and clinical outcomes prediction. The data was obtained from the VitalDB dataset, which is a comprehensive dataset composed of intraoperative bio-signals and clinical information [22]. The dataset includes high-quality bio-signals such as 500 Hz ECG waveform signals from patients who underwent routine or emergency surgery in 10 out of 31 operating rooms in Seoul National University Hospital from August 2016 to June 2017. All the patients got non-cardiac (general, thoracic, urologic, and gynecologic) surgeries. The intraoperative ECG signals were used to calculate different HRV measurements, while clinical information was used as auxiliary features.

To develop our machine learning models, we used a cohort of 6531 cases from the dataset, with an additional selection criterion applied. We extracted the ECG II signals recorded by the SNUADC device for each case. The ECG lead II was used because it has the most obvious characteristics for P and R waves. Cases with an ECG signal duration of less than one hour were excluded. A total of 6064 cases remained for HRV calculation.

### Feature extraction

BioSPPy, a toolbox for bio-signal processing, was used to extract the R wave from each ECG signal. R peaks were then detected, and NN intervals were calculated. To facilitate further analysis, data cleaning was performed using the hrv-analysis package to remove outliers and corrupted data.

After R peaks detection, HRV features were then computed using the pyhrv package [23]. There were 76 effective HRV features computed from each signal, including time domain, frequency, and nonlinear features. Additional 9 clinical features such as age, operation time, and anesthesia time were also included. The feature set used in our study consisted of the HRV features and the clinical features. These features were selected to fully reflect the characteristics of patients under anesthesia. Clinical information provided the patient’s health status and demographic information, while HRV information could quantify the overall changes in heart rate. Clinical and HRV information complemented each other, leading to better patient outcomes predictions. The prediction models were trained with only clinical information, and with both clinical information and HRV features, respectively and separately.

A certain amount of HRV features were missing in some cases due to ECG data quality. We excluded cases with a missing rate more than 20% to reduce the impact of data quality. For cases that remained, missing values were forward filled, which means to fill in the missing value with the previous value. Backward filling and linear interpolation were also tested, and they turned out to have little impact on the results. In addition, outliers were detected and removed.

**Fig. 1:**
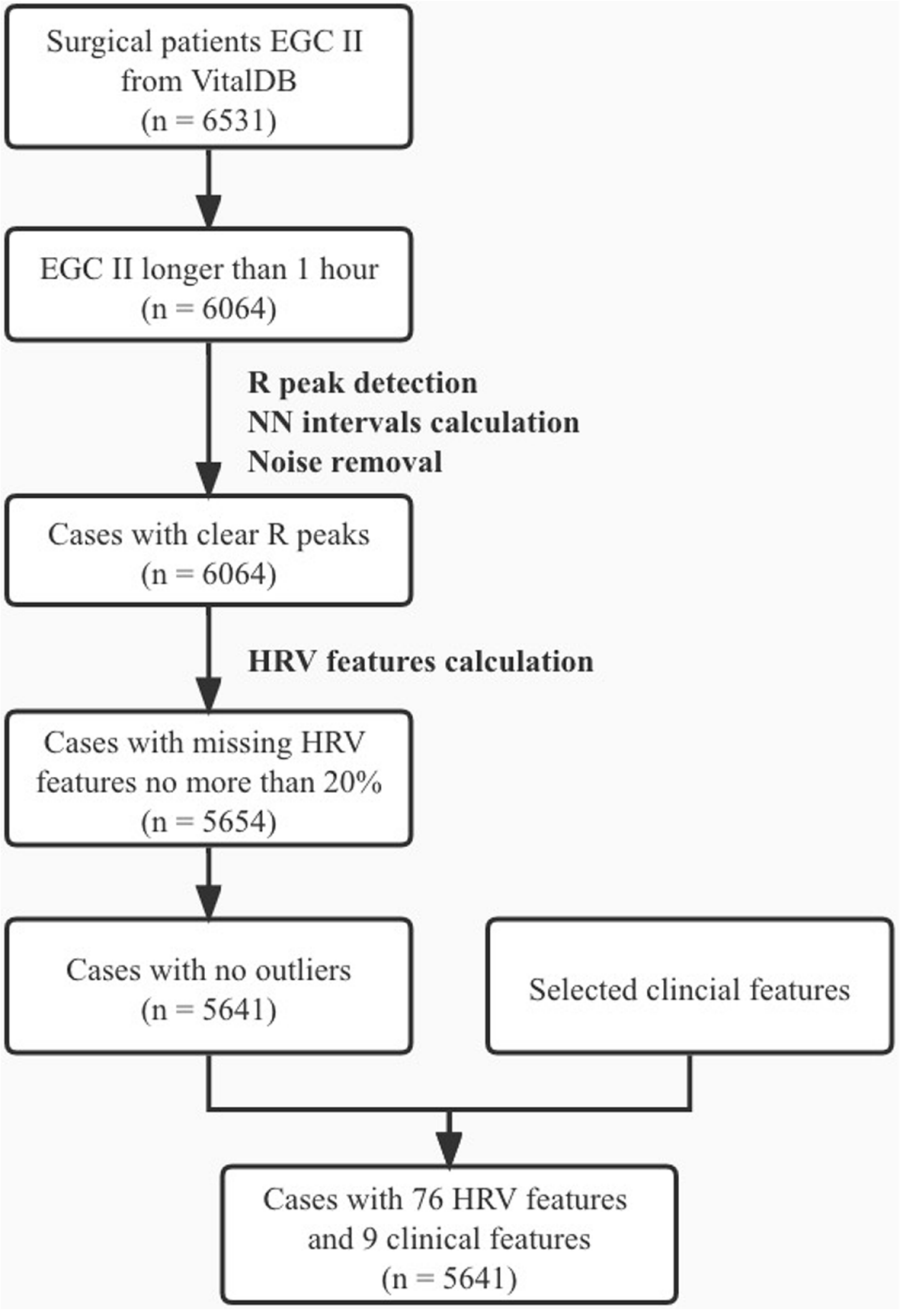
Flow diagram of case selection and inclusion criteria for cases in VitalDB dataset. Certain cases with sufficient duration and clear features were selected. Supplementary clinical features were included

After data filtering, A total of 5641 cases were eligible for analysis. We chose 30-min segment from each case as a feature-extracted target. Preferably, we chose segments with less filled data. 85 features were extracted from each case, of which 76 were HRV features and 9 were clinical features. The complete cohort selection and feature extraction process are shown in the flow diagram of Fig. 1.

### Experiment methods

The primary outcome of this study was the postoperative ICU stay, the secondary outcome was in-hospital mortality, and the total length of hospital stay. The Postoperative ICU stay was transformed into a two-class problem, i.e., whether or not the patients go to ICU after surgery. The Total length of hospital stay was transformed from the continuous values of the hospital stay duration into four intervals of different lengths, leading to a multi-class problem. The in-hospital mortality contains very few death outcomes, resulting in an extreme data imbalance. To deal with the data imbalance problem, SMOTE technique [24] was used to synthesize new samples for the minority class which effectively balances the class distribution. With oversampling of the minority class data, we obtained a more balanced dataset, this approach could help the prediction models to achieve better performance.

In our experiment, we randomly split the dataset with no patient overlap into two: 70% of the cases as the train set, and the remaining 30% of the cases as the test set. We performed five-fold cross-validation on the train set to select the best hyper-parameters for each model. F1-score and AUROC metrics were used to evaluate the model performance. The ROC curve shows the relation between true positive rate (i.e., the fraction of predicted ICU stays that stayed in ICU, also called sensitivity) and false positive rate (the fraction of non-ICU stays but predicted to stay ICU, equals to 1-specificity) under different probability thresholds. The closer the ROC curve is to the upper left, the higher area under the curve and the better discriminatory ability the model could achieve. The area under the curve (AUROC) is an important indicator for evaluating the classifier’s performance.

### Prediction models

Six machine learning models including Logistic Regression (LR), Support Vector Machine (SVM), Random Forest (RF), Gradient Boosting Decision Trees (GBT), Extreme Gradient Boosting (XGB), an ensemble of these five baseline models were developed to predict the three clinical outcomes, which makes up a total of 18 models. Logistic Regression (LR) was used for its low computational time, and robustness to small noise. Support Vector Machines (SVM) are generally not sensitive to noise and outliers since the model focuses on a small set of support vectors in which a hyperplane is constructed from the support vectors to separate between classes, this helps it capture key samples and ignore most other large numbers of redundant samples. Random Forest (RF) has the advantage of processing high-dimensional data, and feature importance can be measured with this model. Gradient Boosting Decision Trees (GBT) were also included because of their flexibility in processing various types of data, namely continuous values and discrete values. Using some robust loss functions, GBT could be very robust to outliers. On this basis, to prevent overfitting, Extreme Gradient Boosting (XGB) was also included. Furthermore, an ensemble of these five models was also tested in our study.

## Results

The characteristics of the 5641 eligible cases are presented in Table 1, some demographic information and HRV features are also included in the table (Percentage values may not add up to 100 due to rounding). Upon counting the characteristics of features, several T-tests were carried out. People with different characteristics were assumed to have the same distribution of ICU stay time in T-tests, while p-values were lower than 0.001 and the assumptions were rejected. Therefore, T-tests proved that people with different characteristics might have different lengths of ICU stay. This test proved that it was possible to predict postoperative clinical outcomes based on distinct characteristics.

**Table 1:** Clinical and HRV characteristics for 5641 patients undergoing non-cardiac surgery

Features	**Characteristic**	**Count (%)**	***p*-value**
**Gender**			< 0.001
	male	2877 (51.0)	
	female	2764 (49.0)	
**Age (years)**			< 0.001
	0–19	96 (1.7)	
	20–39	684 (12.1)	
	40–59	2288 (40.6)	
	60–79	2390 (42.4)	
	80 +	183 (2.2)	
**Anesthesia time (hours)**			< 0.001
	0–2	1656 (29.4)	
	3–4	2270 (40.2)	
	5–6	1221 (21.6)	
	7 +	494 (8.8)	
**Average heart rate**			< 0.001
	0–59	552 (9.8)	
	60–69	1516 (26.9)	
	70–79	1705 (30.2)	
	80–99	1469 (26.0)	
	100 +	399 (7.1)	
**Relative FFT LF Power**			< 0.001
	0–19	993 (17.6)	
	20–39	2964 (52.5)	
	40–59	1648 (29.2)	
	60 +	36 (0.6)	
**DFA** ${\boldsymbol{\alpha }}_{1}$			< 0.001
	0–1	383 (6.8)	
	1–1.2	1728 (30.6)	
	1.2–1.4	3046 (54.0)	
	1.4 +	484 (8.6)	
**Postoperative ICU stay**			/
	No	4658 (81.0)	
	Yes	1073 (19.0)	
**In-hospital mortality**			/
	No	5589 (99.1)	
	Yes	52 (0.9)	
**Total length of hospital stays (days)**			/
	0–3	1103 (19.6)	
	4–6	1371 (24.3)	
	7–9	1412 (25.0)	
	10 +	1755 (31.1)	

Among the 5641 unique cases, 2877 (51.0%) were male. 4678 (83.0%) cases had ages over 40 while only 183 (2.2%) cases had ages over 80. For the outcome variables, 1073 (19.0%) cases stayed in ICU after surgery, and only 52 (0.9%) cases died in the hospital compared to 5589 (99.1%) cases did not die in the hospital. The proportion of the four classes in the total length of hospital stay outcome is relatively balanced compared to the other two outcomes, ranging from 1103(19.6%) to 1755(31.1%).

**Fig. 2:**
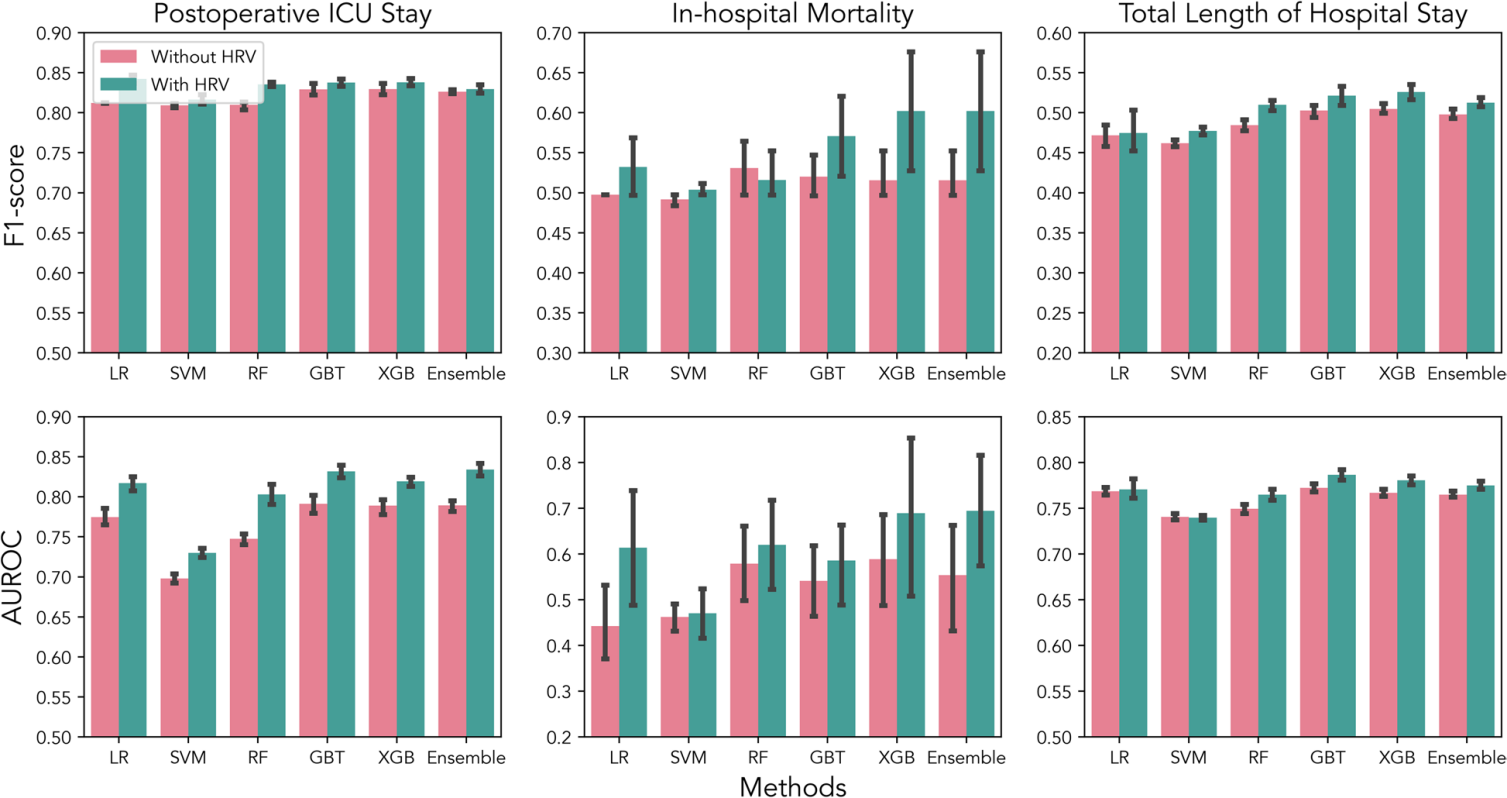
F1-score and AUROC of all 18 models on 3 clinical outcomes prediction with mean and 95% confidence interval reported. In most cases, using both clinical information and HRV features could significantly improve the performance of the baseline models

In the final test set, postoperative ICU stay, in-hospital mortality, and total length of hospital stay were predicted. The F1-score and AUROC of all 18 models on the three clinical outcome predictions are computed and shown in Fig. 2. The F1-score is the harmonic mean of the precision and recall, which symmetrically represents both precision and recall in one metric. The AUROC is the area under the ROC curve, which shows the performance of a classification model at all possible thresholds. Using only clinical information (without HRV features, which are No.77 to No.85 features in Table S1 in supplementary files), the highest AUROC that all models could achieve were 0.79, 0.58, 0.76 for postoperative ICU stay, in-hospital mortality, and total length of hospital stay, respectively. While using both clinical information and HRV features (all 85 features in Table S1 in supplementary files), the highest AUROC that the models could reach were 0.83, 0.70, and 0.76 for the three clinical outcomes, respectively. In almost all cases, the presence of HRV features significantly improves the performance of the baseline models (both AUROC and F1- score). The only exception was the result from the in-hospital mortality prediction where we obtained an F1-score of 0.53 with clinical information compared to 0.51 using both clinical and HRV features. These results suggested that HRV during anesthesia can be useful for predicting postoperative clinical outcomes.

**Fig. 3:**
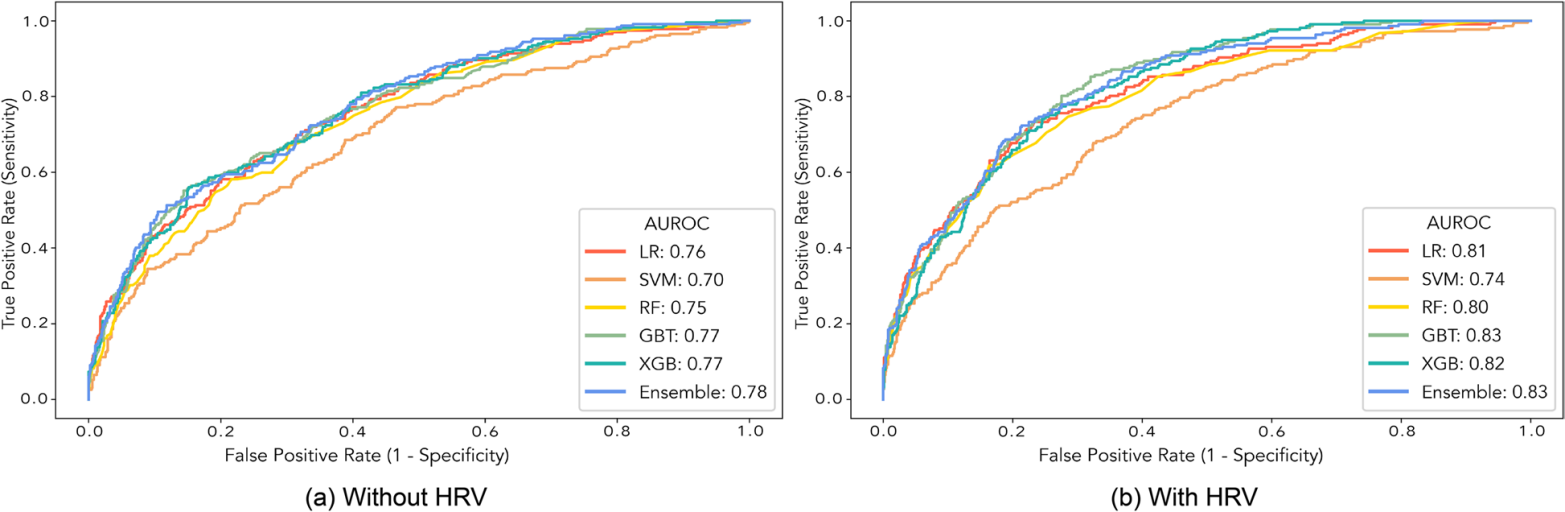
ROC curves for postoperative ICU stay prediction. Among all six models, the true positive rate increased while HRV features were included, which suggests the effectiveness of HRV features in postoperative ICU stay prediction

The ROC curves of different models for postoperative ICU stay prediction are shown in Fig. 3. All six models achieved similar results, in which AUROC without HRV features could reach up to 0.78, and that can be increased to 0.83 when including HRV features, which suggests the effectiveness of HRV features in postoperative ICU stay prediction.

To explore the simplified parameters of intraoperative HRV during anesthesia for poor prognosis, we performed a subgroup analysis. We found that patients with an average heart rate higher than 70 (0.255 vs. 0.230,$p-value < 0.001$) (Fig. S1), low-frequency power (LFP) lower than 33 (0.275 vs. 0.238 $p-value < 0.001$) (Fig. S2), short-term fluctuation DFA ${\alpha }_{1}$ lower than 0.95 (0.319 vs. 0.254, $p-value < 0.001$) (Fig. S3), during anesthesia had a significantly higher risk of entering the ICU after surgery. Cut-off values of the parameters referred to the classical research, namely the Framingham Heart Study [13] and BEAUTIFUL analysis [25]. Results were consistent with the classical research.

## Discussion

### HRV measurements

Various types of measurements will be considered when selecting appropriate features. HRV measurements can be analyzed in the time domain, frequency domain, and non-linear measures. Time domain analysis quantifies statistics from the overall time intervals between normal heartbeats. Frequency domain measurements calculate the power spectrum from the R-R intervals. Non-linear measures quantify the overall variability into useful nonlinear prosperities using various techniques such as invariant methods, informational entropy methods, and geometrical methods.

Due to easy settings, time domain measures have been widely used. The most commonly used measures of time domain metrics include NN intervals, SDNN, SDANN, RMSSD, NN50, and pNN50. NN intervals refer to the intervals between normal R peaks, therefore NN intervals and R-R intervals are synonymous. SDNN is the standard deviation of the NN intervals; it measures the variation of the NN intervals which reflects the combined influence of both SNS and PNS activity [26]. Similar to SDNN, SDANN computes the standard deviation using the average of NN intervals from each 5-min segment of a long recording. RMSSD is the root mean square of successive NN interval differences; it reflects beat-to-beat variance in short-term HRV. The number of consecutive NN intervals that differs by more than 50 ms is known as NN50, and pNN50 is the percentage of NN50 over the entire NN series.

Frequency domain measurements utilize Fast Fourier Transform to decompose HRV signal energy into various frequency bands; the four most widely used frequency ranges are ultra-low-frequency (ULF, $\le 0.003 Hz$), very-low-frequency (VLF, $0.0033- 0.04 Hz$), low-frequency (LF, $0.004-0.15 Hz$), and high frequency (HF, $0.15-0.40 Hz$). The signal energy within a frequency band can be expressed in absolute or relative power, which is measured as milliseconds squared per hertz ($m{s}^{2}/Hz$) and normal units (nu), respectively. The ULF band indicates fluctuation in consecutive normal beats intervals. The VLF band captures rhythms with periods between 25 and 300 s. The LF and HF bands are influenced by breathing, and LF/HF (ratio of LF to HF) may be used to measure the proportion of the SNS and PNS activity under a controlled state [27, 28].

Non-linear measurements offer a tool to quantify the complexity mechanisms of HRV, which cannot be effectively modeled under stationary requirements and linear assumptions. Poincaré plot is one of the geometrical methods, it presents graphical plots in which a scatter plot is constructed from each NN interval plotted against its successive NN intervals. Several non-linear features can be obtained by fitting an ellipse to the plotted points including the standard deviation of the major and minor axes (SD1 and SD2, respectively), SD2/SD1 ratio, and area of the Poincaré ellipse. Entropy methods such as sample entropy allow us to quantify the complexity or irregularity of a series of RR intervals, it has the advantage of being less biased and consistent for very short time series. Detrended fluctuation analysis (DFA) is a technique for detecting the long-term and short-term correlation of NN intervals. This method was proposed specifically to handle non-stationary characteristics in long-term HRV series. The short-term fluctuation denoted as ${\alpha }_{1}$ reflects the baroreceptor reflex, while the long-term fluctuation denoted as ${\alpha }_{2}$ indicates the regulatory mechanisms that control alternating activities of the beat cycle.

Different measurements indicate different characteristics of the case. Therefore, HRV measurements analyzed in the time domain, frequency domain, and non-linear measures are all taken into consideration, as well as some clinical features. 76 HRV features and 9 clinical features are used in the experiment, and details are described in the supplementary files.

### Importance analysis

We conducted experiments to verify the prognostic values of the intraoperative HRV using various machine learning models, the results suggested that HRV is effective for postoperative clinical outcome predictions. Notice that the 95% confidence interval of in-hospital mortality outcome predictions was relatively large compared to the confidence interval of the other two outcome predictions, this is due to the extreme class imbalance in the data in which the models had high uncertainty in their predictions. In addition, we observed that the in-hospital mortality prediction had the most performance increases when using both clinical and HRV features. Although SMOTE technique was applied in both settings (with or without HRV features), using only clinical information appeared to achieve very poor results, this showed that HRV features played a significant role in class imbalance setting, and when clinical information alone is insufficient for predicting related clinical outcomes, which is, in this case, the in-hospital mortality.

**Fig. 4:**
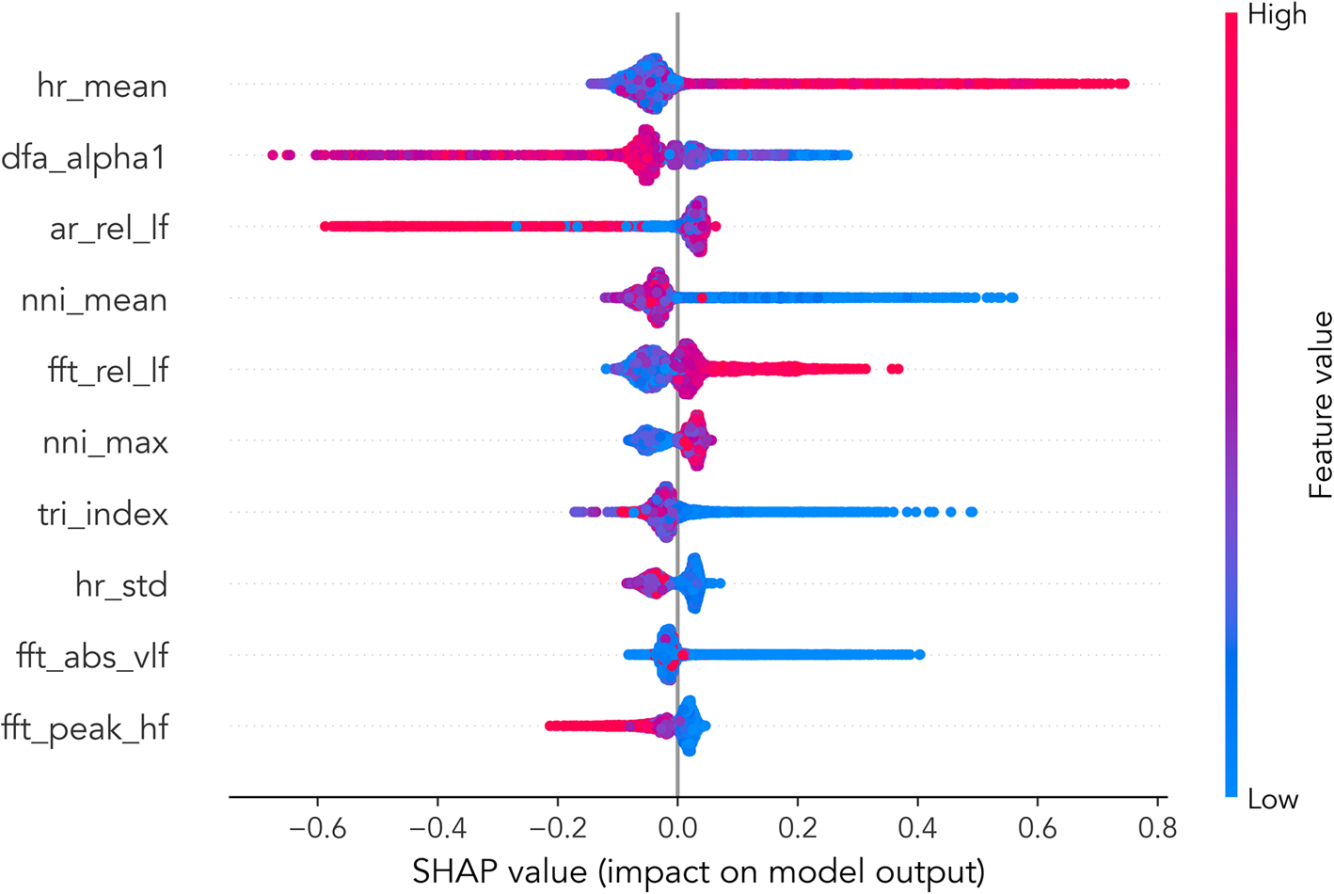
Top 10 HRV features with the highest SHAP value. The most important HRV features mainly include heart rate, frequency domain low-frequency intensity and short-term fluctuations. The results could be supported by clinical practice. Therefore, these features can be used as diagnostic basis

We investigated into feature importance of HRV and used it as theoretical support to analyze the classification decisions of the models. We computed the importance of HRV features using the SHAP value method [29] for postoperative ICU stay prediction. The SHAP value method quantifies contribution that each feature brings to the prediction made by the model. The top 10 HRV features given by the SHAP value method are shown in Fig. 4. The most important HRV features mainly include heart rate (mean of heart rate and NN interval), frequency domain low-frequency intensity, and short-term fluctuations DFA ${\alpha }_{1}$. The result from the SHAP value plot in Fig. 4 showed that mean of heart rate (*hr_mean*), relative energy of low frequency after FFT (*fft_rel_lf*), and max NN interval (*nni_max*) were associated with a higher risk of postoperative ICU stay when the feature values are increased. Contrarily, increases in DFA ${\alpha }_{1}$(*dfa_alpha1*), mean of NN interval (*nni_mean*), triangular index measurement (*tri_index*, equals the number of all NN intervals divided by the maximum of the density distribution), the standard deviation of heart rate (*hr_std*), absolute energy of very low frequency after FFT (*fft_abs_vlf*), the peak of high-frequency HRV after FFT (*fft_peak_hf*) were found to be associated with lower risk of postoperative ICU stay.

Regarding the heart rate, it was shown that patients with a heart rate higher than 70 could increase the risk of cardiovascular diseases [25]. To understand the outcome risks of each subgroup, we conducted a subgroup analysis that used the same threshold of 70 for average heart rate to divide cases into two subgroups. Our finding was consistent with the retrospective study mentioned earlier (Fig. S1).

For low-frequency power (LFP) in the frequency domain feature, it was reported that lower LFP will lead to a higher risk of death from all causes [17]. In this subgroup analysis, we divided LFP evenly into three subgroups. We showed that lower LFP was associated with a higher risk of entering the ICU after surgery, which was also consistent with the previous studies (Fig. S2).

For short-term fluctuation DFA ${\alpha }_{1}$, it was found that when short-term fluctuation is low, i.e., DFA ${\alpha }_{1}$ lower than 0.95, the patient’s survival probability will be significantly reduced [30]. Similarly, we used the same threshold of 0.95 and conducted a subgroup analysis for short-term fluctuation. The result suggested that patients with DFA ${\alpha }_{1}$ lower than 0.95 would have a significantly higher risk of entering the ICU after surgery (Fig. S3).

From a medical perspective, heart rate is the most direct manifestation of heart health. Low-frequency power reflects a mixture of sympathetic and parasympathetic activity of the heart. Short-term fluctuation represents a response to the transient change in blood pressure, which can indicate the autonomic regulation of heart rate. Therefore, those features can reflect prognosis.

### Strengths and limitations

Our experiment demonstrated that the use of HRV could potentially increase the predictive performance across all trained models. Multiple commonly used machine learning models were adopted, which formed a comprehensive experiment. Using machine learning methods, we analyzed clinical problems more quantitatively, which gave an understandable explanation of the importance of HRV during anesthesia.

Acknowledge that there is still room for improvement, we hope to expand the scope of application, from low-risk to other high-risk diseases. Also, due to data limitations, we train and verify the data in the same database. Therefore, some other data should be introduced for verification. In addition, we hope that HRV analysis would be applied to real-world clinical applications to provide target therapy for patients with abnormal HRV.

## Conclusion

In this study, we developed multiple machine learning models for postoperative clinical outcomes prediction among patients undergoing surgery with anesthesia using clinical and HRV data collected from the VitalDB database. The presence of HRV features significantly improves the performance of the baseline models, the results suggested that HRV during anesthesia is feasible for predicting postoperative clinical outcomes. We identified and analyzed the most effective features as well.

## Supplementary Information

**Additional file 1. **

## Data Availability

The datasets analysed during the current study are available in the VitalDB repository, https://vitaldb.net/dataset/?query=api.
