# Supplementary material for: The prognostic value of intraoperative HRV during anesthesia in patients presenting for non-cardiac surgery
Source: BMC Anesthesiol. 2023 May 9;23:160. doi: 10.1186/s12871-023-02118-9 (PMC10169477; doi:10.1186/s12871-023-02118-9)
Supplement: Supplementary file 1 — Additional file 1. [file 12871_2023_2118_MOESM1_ESM.pdf]

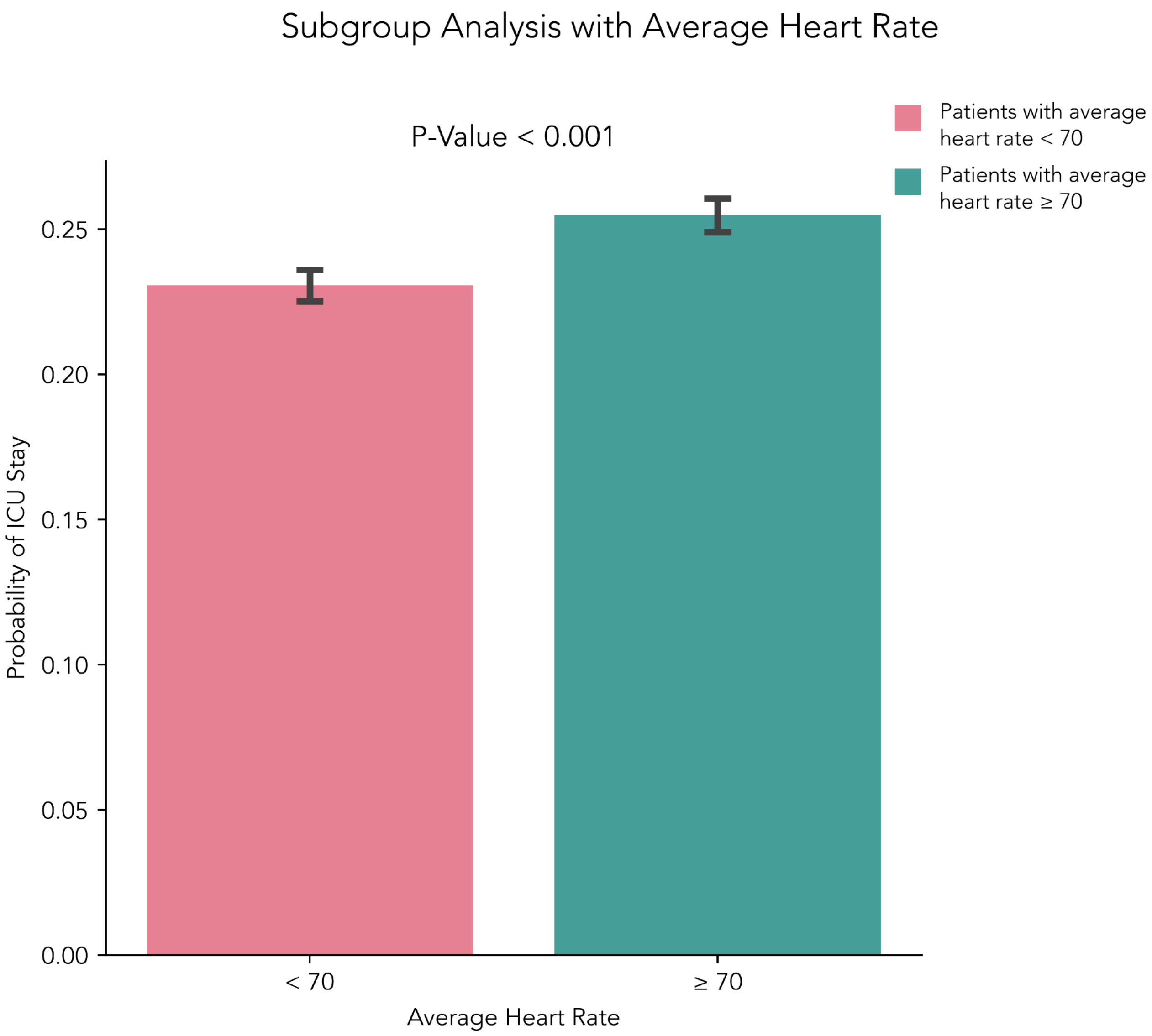


Fig S1. Subgroup analysis with average heart rate. Patients with average heart rate higher than 70 are likely with higher risk of entering ICU after surgery.

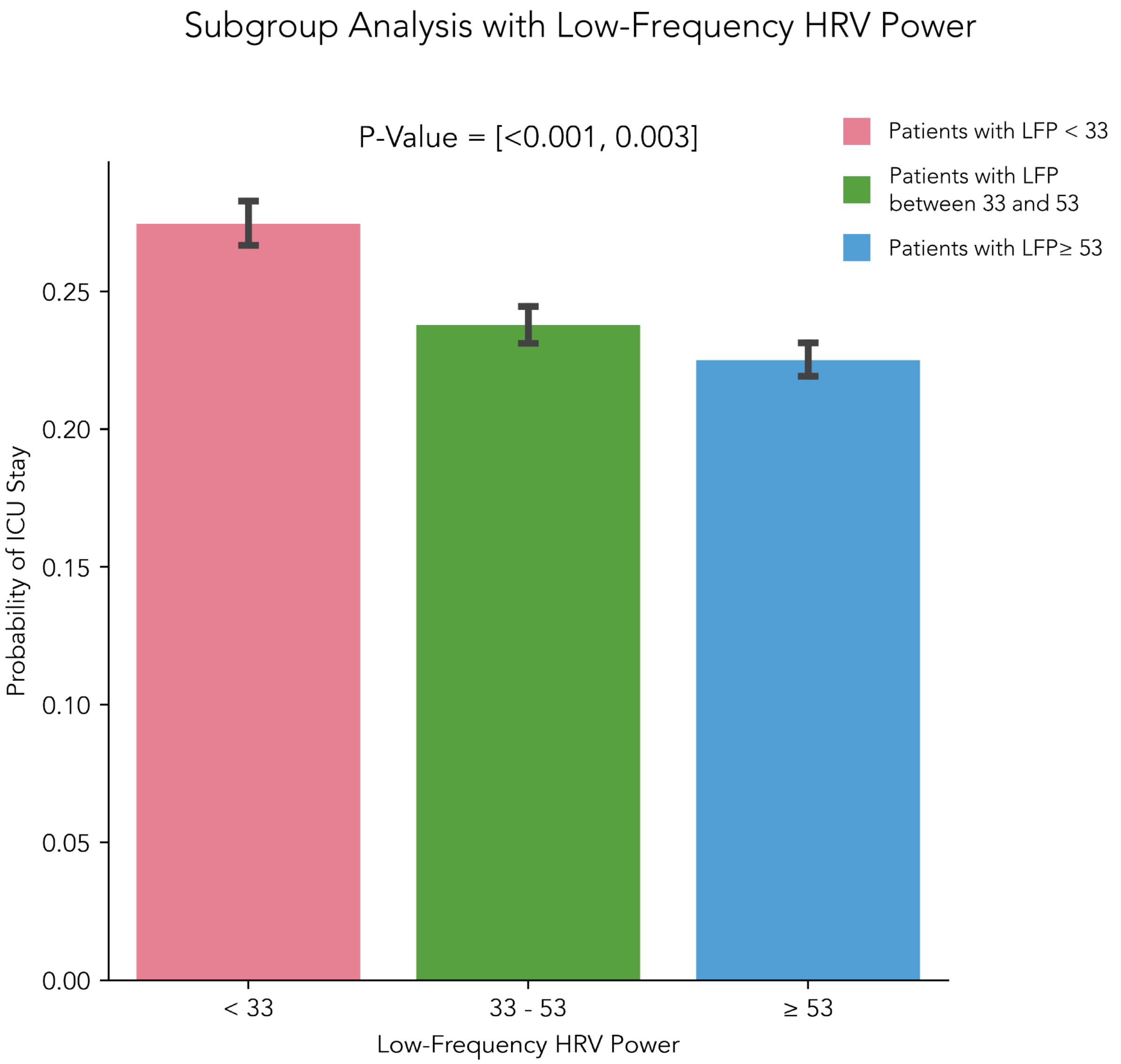


Fig S2. Subgroup analysis with low-frequency HRV power. Patients with low-frequency power (LFP) < 33 are likely with higher risk of entering ICU after surgery.

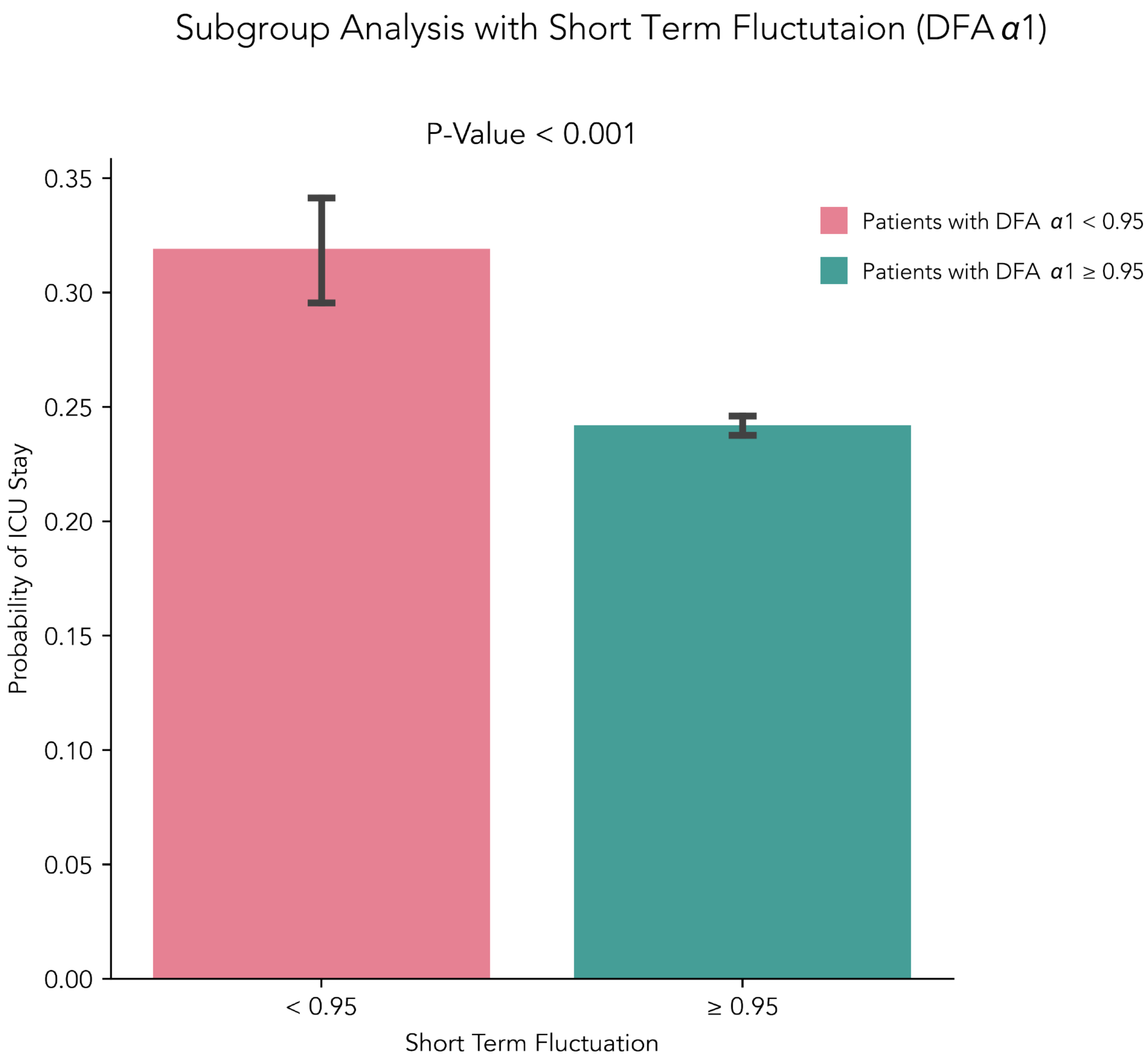


Fig S3. Subgroup analysis with short term fluctuation. Patients with short-term fluctuation DFA $\alpha 1 < 0.95$ are likely with higher risk of entering ICU after surgery.

Table S1. Extracted features in each case

| No | **Feature abbreviation** | **Brief explanation** |
| --- | --- | --- |
| HRV features | | |
| 1 | nni_counter | related to NN intervals |
| 2 | nni_mean | |
| 3 | nni_min | |
| 4 | nni_max | |
| 5 | hr_mean | related to heart rate features |
| 6 | hr_min | |
| 7 | hr_max | |
| 8 | hr_std | |
| 9 | nni_diff_mean | related to NN interval differences |
| 10 | nni_diff_min | |
| 11 | nni_diff_max | |
| 12 | sdnn | related to standard deviation of a NN interval series |
| 13 | sdnn_index | |
| 14 | sdann | |
| 15 | rmssd | root mean of squared NNI differences |
| 16 | sdsd | standard deviation of NNI differences |
| 17 | pnn50 | related to the ratio of NN more than 50/20ms |
| 18 | pnn20 | |
| 20 - 22 | fft_peak_0 - 2 | related to FFT power spectral density |
| 23 - 25 | fft_abs_0 - 2 | |
| 26 - 29 | fft_rel_0 - 2 | |
| 29 - 31 | fft_log_0 - 2 | |
| 32 - 33 | fft_norm_0 - 1 | |
| 34 | fft_ratio | |
| 35 | fft_total | |
| 36 | fft_nfft | |
| 37 - 39 | lomb_peak_0 - 2 | related to Lomb-Scargle power spectral density |
| 40 - 42 | lomb_abs_0 - 2 | |
| 43 - 45 | lomb_rel_0 - 2 | |
| 46 - 48 | lomb_log_0 - 2 | |
| 49 - 50 | lomb_norm_0 - 1 | |
| 51 | lomb_ratio | |
| 52 | lomb_total | |
| 53 | lomb_fft | |
| 54 - 56 | ar_peak_0 - 2 | related to autoregressive power spectral density |
| 57 - 59 | ar_abs_0 - 2 | |
| 60 - 62 | ar_rel_0 - 2 | |
| 63 - 65 | ar_log_0 - 2 | |

| No | **Feature abbreviation** | **Brief explanation** |
| --- | --- | --- |
| 66 | ar_norm_0 - 1 | |
| 67 | ar_ratio | |
| 68 | ar_total | |
| 69 | ar_fft | |
| 70 | sd1 | related to Poincare |
| 71 | sd2 | |
| 72 | sd_ratio | |
| 73 | ellipse_area | |
| 74 | sampen | sample entropy |
| 75 | dfa_alpha1 | related to detrended fluctuation analysis (DFA) |
| 76 | dfa_alpha2 | |
| Clinical features | | |
| 77 | ane_time | related to basic patient information |
| 78 | op_time | |
| 79 | age | |
| 80 | sex | |
| 81 | height | |
| 82 | weight | |
| 83 | bmi | |
| 84 | emop | |
| 85 | ane_type | |
